# Fetal first trimester growth is not associated with kidney outcomes in childhood

**DOI:** 10.1007/s00467-016-3537-8

**Published:** 2016-10-27

**Authors:** Hanneke Bakker, Romy Gaillard, Albert Hofman, Irwin K. Reiss, Eric A. P. Steegers, Vincent W. V. Jaddoe

**Affiliations:** 1 The Generation R Study Group (Na-29-15), Erasmus University Medical Center, Box 2040, 3000 CA Rotterdam, The Netherlands; 2 Department of Epidemiology, Erasmus University Medical Center, Rotterdam, The Netherlands; 3 Department of Pediatrics, Erasmus University Medical Center, Rotterdam, The Netherlands; 4 Department of Epidemiology, Harvard T.H. Chan School of Public Health, Boston, MA USA; 5 Department of Obstetrics & Gynaecology, Erasmus University Medical Center, Rotterdam, The Netherlands

**Keywords:** Fetal development, Crown–rump length, Kidney function, Childhood, Kidney volume

## Abstract

**Background:**

Impaired fetal growth is associated with increased risks of kidney diseases in later life. Because human development rates are highest during the first trimester, this trimester may be a particularly critical period for kidney outcomes. We have therefore examined the association of fetal first trimester growth with kidney outcomes in childhood.

**Methods:**

This study was embedded in a prospective population-based cohort study among 1176 pregnant women and their children. We used fetal first trimester crown–length as the growth measure among mothers with a regular menstrual cycle and a known first day of the last menstrual period. At the childhood age of 6 (median 5.7–6.8) years, we measured combined kidney volume, microalbuminuria and estimated glomerular filtration rate (eGFR) based on serum creatinine and cystatin C concentrations.

**Results:**

No consistent associations of fetal first trimester crown–rump length with childhood combined kidney volume, eGFR and microalbuminuria were observed. Compared to children with a fetal first trimester crown–rump length in the highest quintile, those in the lowest quintile had a larger childhood combined kidney volume (difference 5.32 cm^3^, 95 % confidence interval 1.06 to 9.57), but no differences in kidney function.

**Conclusion:**

Our results do not support the hypothesis that fetal first trimester growth restriction affects kidney size and function in childhood. Further studies are needed to focus on critical periods in early life for kidney function and disease in later life.

**Electronic supplementary material:**

The online version of this article (doi:10.1007/s00467-016-3537-8) contains supplementary material, which is available to authorized users.

## Introduction

Chronic kidney disease (CKD) may originate in the earliest phase of life [1]. It has been hypothesized that adverse environmental exposures in utero may lead to kidney developmental adaptations, including lower nephron numbers leading to glomerular hyperfiltration [2–4]. These adaptations may subsequently progress to glomerulosclerosis, impaired kidney function and increased risks of CKD in adulthood [5, 6]. This hypothesis is supported by observational studies showing associations of preterm birth or small-size for gestational age at birth with smaller kidneys and increased risk of kidney disease later in life [7–9]. In an earlier study, our group observed that decreased second and third trimester fetal growth and lower infant growth rates are associated with smaller kidneys in childhood [8]. We also noted that decreased second and third trimester fetal weight growth is associated with lower kidney function in childhood [8]. To date, little is known about the influence of first trimester fetal development on kidney outcomes later in life. Nephrogenesis starts around the eighth week of gestation and ceases around 36 weeks of gestation [10]. Because of the relatively high developmental rates, first trimester might also be a critical period for kidney development. Fetal first trimester crown–rump measurement is often used to determine gestational age in obstetric care practice which suggests there is no variation in early fetal growth [11]. However, we have previously shown that in women with a regular cycle and a known first day of their menstrual period, fetal first trimester crown–rump length can also be used to assess differences in embryonic growth rate [11, 12].

We assessed, in a population-based prospective cohort study involving 1176 mothers and their children, the associations between fetal first trimester crown–rump length and kidney outcomes in childhood. Kidney outcomes included combined kidney volume, estimated glomerular filtration rate (eGFR) based on creatinine and cystatin C blood levels and microalbuminuria. Since kidney function tracks from childhood into adulthood, subclinical variations at young age might already be associated with renal impairment in later life [7, 8].

## Methods

### Design and study population

**Fig. 1:**
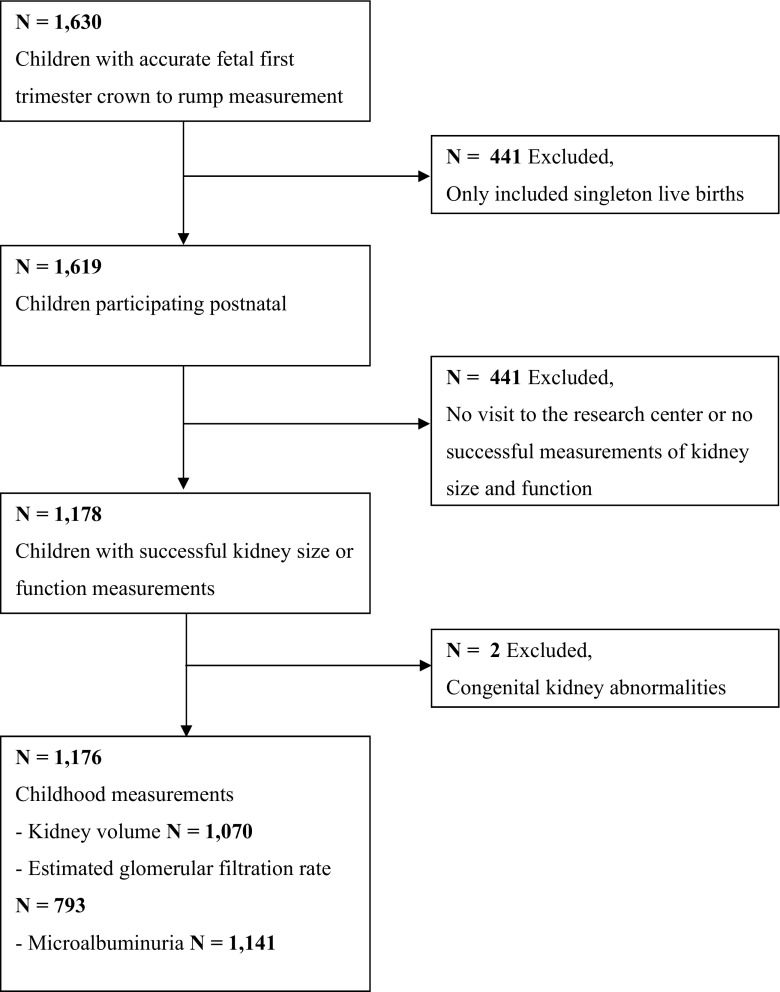
Flowchart of study design showing the inclusion of participants in the analyses

This study was embedded in the Generation R Study, a population-based prospective cohort study from fetal life onwards in Rotterdam, the Netherlands [13]. As previously described, inclusion in the study was aimed at women in the early stages of pregnancy but enrollment was allowed until birth [14]. Of the total study cohort of 9901 mothers, 1630 mothers had an available fetal first trimester crown–rump measurement within the range of 10 weeks 0 days to 13 weeks 6 days and had a reliable gestational age estimate based on the last menstrual period and a regular menstrual cycle, and were therefore eligible for enrollment in this study [12]. We included only mothers who gave birth to a singleton live-born child (*n* = 1619). In total, 1176 mothers and children were included in the detailed follow-up measurements at the age of 6 years. Blood and urine samples for kidney function measurements were available in 793 (67 %) and 1141 (98 %) children, respectively. Missing blood samples were mainly due to lack of consent. A flowchart of the study design is given in Fig. 1. Differences in subject characteristics between children with and without blood samples are shown in Electronic Supplemental Material (ESM) Table 1. There were no differences in fetal first trimester crown–rump length between children with and without blood sample measurements.

### Fetal first trimester crown–rump length

Fetal first trimester crown–rump length measurements were carried out in the gestational age range of 10 week 0 days to 13 weeks 6 days in a true mid-sagittal plane with the genital tubercle and the fetal spine longitudinally in view on the ultrasound scan [12]. Intraclass correlation coefficients were ≥0.995 [15]. Information on the first day of the last menstrual cycle was obtained from the referring letter from the midwife or hospital and was confirmed at enrollment [12]. Mothers gave additional information on the regularity and duration of the menstrual cycle at the ultrasound visit. Gestational age adjusted standard deviation scores (SDS) for first trimester crow–rump length were constructed as described by Mook-Kanamori et al. [12].

### Childhood kidney outcomes

The left and right kidney biometrics of each child at the median age of 6 (90 % range 5.7–6.8) years were measured as described previously [8, 16]. The child was awake and calm in a standardized prone position during the ultrasound examination. Maximal bipolar kidney length, width and depth were measured; kidney width and depth were measured at the level of the hilum. The cross-sectional area in which the kidney appeared symmetrically round at its maximum width was used. Kidney volume was calculated using the equation of an ellipsoid: volume (cm^3^) = 0.523 × length (cm) × width (cm) × depth (cm) [17]. Combined kidney volume was calculated by summing right and left kidney volume. Previously reported intra-observer and inter-observer correlation coefficients were used [18].

Blood samples were drawn by antecubital venipuncture. Serum creatinine levels were measured using an enzymatic method on a Cobas c502 analyzer (Roche Diagnostics GmbH, Mannheim, Germany), and serum cystatin C levels were measured using a particle-enhanced immunoturbidimetric assay on Cobas c702 analyzer (Roche Diagnostics GmbH). Intra- and inter-assay coefficients were used a described previously [19]. The eGFR was calculated according to the revised Schwartz 2009 formula [20]: eGFR_creat_ = 36.5 × [height (cm)/serum creatinine (μmol/l] [20]. The eGFR based on cystatin C levels according to the Zappitelli formula [21] was also calculated: eGFR_Cyst_ = 75.94/[CysC^1.17^] [16]. Urine creatinine (mmol/l) and urine albumin (mg/l) levels were determined on a Beckman Coulter AU analyzer (Beckman Coulter Inc., Brea, CA); creatinine levels were measured according to the Jaffe method. The albumin:creatinine ratio was calculated, and microalbuminuria was defined as an albumin:creatinine ratio of between 2.5 and 25 mg/mmol for boys, and between 3.5 and 25 mg/mmol for girls [22].

### Covariates

Information on maternal age, pre-pregnancy weight, parity, ethnicity, educational level, smoking during pregnancy, alcohol consumption during pregnancy, folic acid supplementation during pregnancy and breastfeeding was obtained by questionnaires and registries. Maternal height was measured without shoes, and pre-pregnancy body mass index (BMI) was calculated. Date of birth, infant sex and birth weight were obtained from midwife and hospital registries. At the age of 6 years, child height and weight were measured without shoes and heavy clothing, and body surface area (BSA) was calculated using the DuBois formula (BSA = weight (kg) ^0.425^ × height (cm) ^0.725^ × 0.007184) [23].

### Statistical analyses

First, we analyzed the associations of fetal first trimester crown–rump length with childhood kidney outcomes (kidney volume, eGFR based on creatinine and cystatin C levels and microalbuminuria) by using multiple linear and logistic regression models. We used first trimester crown–rump length SDS as continuous variables and as quintiles to explore non-linear associations. Sensitivity analyses were performed using tertiles of fetal first trimester growth. To explore the associations of lower and higher growth as compared to average growth, we compared the lowest and the highest tertile with the middle tertile. The fully adjusted model was adjusted for maternal age, educational level, ethnicity, parity, pre-pregnancy BMI, smoking during pregnancy, alcohol consumption during pregnancy, folic acid supplement use, breastfeeding and current BSA. To take into account body composition, analyses focused on total kidney volume were indexed for BSA. Analyses focused on eGFR were not adjusted for childhood BSA since height is included in the Schwartz 2009 formula [20]. Potential confounders were based on their associations with kidney outcomes or a change in the effect estimate of >10 %. To reduce the possibility of potential bias due to missing data, we imputed missing data of the fetal, child and maternal covariates with five imputations and analyzed these datasets together [24]. Additional information on the imputation procedure is given in the ESM. All statistical analyses were performed using the Statistical Package for the Social Sciences version 21.0 for Windows (IBM Corp., Armonk, NY).

## Results

### Subject characteristics

Maternal, fetal and child characteristics are shown in Table 1. Mean fetal first trimester crown–rump length was 61.1 (SD 11.4) mm at a median gestational age of 12.4 (90 % range 11.0-13.7) weeks. At the median age of 6.0 (90 % range 5.7-6.8) years, mean combined kidney volume was 119.7 (SD 22.0) cm^3^, creatinine-based eGFR was 119.4 (SD 15.4) ml/min per 1.73 m^2^ and cystatin C based eGFR was 102.8 (SD 15.9) ml/min/1.73 m^2^. Microalbuminuria was present in 7.0 % of all children. Correlations between total kidney volume and BSA-related kidney volume with eGFR based on creatinine and cystatin levels are given in ESM Tables 4 and 5. In total 117 children were born with a small size for gestational age at birth (<10 %) and 54 (4.6 %) children were born preterm (<37 weeks).

**Table 1:** Maternal and child characteristics

Maternal and child characteristics (*N* = 1176)	Values
Maternal characteristics	
Age (years)	31.8 (22.8–38.1)
Height (cm)	168.8 (SD 7.1)
Pre-pregnancy weight (kg)	67.0 (SD 11.8)
Pre-pregnancy body mass index (kg/m^2^)	23.5 (SD 3.9)
Parity, nulliparous	715 (60.8 %)
Ethnicity	
European	853 (72.5 %)
Non -European	323 (27.5 %)
Educational level	
No higher education	525 (44.6 %)
Higher education	651 (55.4 %)
Smoking	
Non-smoking	881 (74.9 %)
Continued smoking	295 (25.1 %)
Folic acid supplement use	
No use	156 (13.3 %)
First 10 weeks use	376 (32.0 %)
Preconception use	644 (54.8 %)
Fetal characteristics	
Gestational age at fetal crow–rump length (weeks)	12.4 (11.0–13.7)
First trimester fetal crown–rump length (mm)	61.1 (SD 11.4)
Birth and infant characteristics	
Males	570 (SD 48.5)
Gestational age (weeks)	40.1 (37.1-42.0)
Birth weight (g)	3,459.2 (SD 549.9)
Breastfeeding	
No	92 (7.8 %)
Yes	1084 (92.2 %)
Child characteristics	
Age (years)	6.0 (5.7–6.8)
Height (cm	119.0 (SD 5.5)
Weight (kg)	22.9 (SD 3.7)
Body mass index (kg/m^2^)	16.1 (SD 1.7)
Kidney volume combined ( cm^3^)	119.7 (SD 22.0)
eGFR (Schwartz, creatinine based) (ml/min per 1.73 m^2^)	119.4 (SD 15.4)
eGFR (Zappitelli, cystatin C based), (ml/min per 1.73 m^2^)	102.8 (SD 15.9)
Microalbuminuria	82 (7 %)

Values in the table are given as the mean with the standard deviation (SD) in parenthesis, as the median with the 90 % range in parenthesis or as the number of subjects with the percentage in parenthesis

eGFR, Estimated glomerular filtration rate

### Fetal first trimester crown–rump length and kidney outcomes in childhood

Table 2 presents the analyses focused on the associations between fetal first trimester crown–rump length and kidney volume and function. Fetal first trimester crown–rump length SDS was not associated with kidney volume and eGFR. There was also no association of fetal first trimester crown–rump length SDS with the risk of microalbuminuria (all* p* values > 0.05).

**Table 2:** Fetal first trimester growth quintiles and childhood kidney volume and function in the 1176 children enrolled in the study

Crown–rump quintiles in standard deviation scores	BSA-adjusted combined kidney volume (cm^3^)	GFR_creat_ (ml/min per 1.73 m^2^)^a^	GFR_Cys_ (ml/min per 1.73 m^2^)^a^	Microalbuminuria (mg/mmol) (OR)
1 (*N* = 238)	5.32 (1.06, 9.57)*	0.24 (−3.33, 3.80)	−0.67 (−4.47, 3.14)	0.60 (0.25, 1.44)
2 (*N* = 234)	−0.88 (−5.14, 3.38)	−0.34 (−3.93, 3.26)	−2.69 (−6.53, 1.15)	0.92 (0.42, 2.02)
3* (N* = 234)	0.42 (−0.85, 4.68)	−0.26 (−3.83, 3.31)	0.33 (−3.48, 4.14)	1.13 (0.52, 2.44)
4 (*N* = 237)	0.54 (−3.75, 4.83)	1.04 (−2.54, 4.64)	−1.11 (−4.96, 2.73)	1.41 (0.66, 2.96)
5 (*N* = 233)	Reference	Reference	Reference	Reference
*p* value for trend	0.10	0.58	0.83	0.51

*p value < 0

Values in table are regression coefficients with the 95 % confidence interval in parenthesis and reflect the difference in childhood kidney outcomes between first-trimester crown–rump length (Quintile 5 is the reference group) The model is adjusted for duration of last menstrual cycle, sex and age of child at time of outcome measurements, maternal age, educational level, ethnicity, parity, pre-pregnancy body mass index, smoking during pregnancy, alcohol consumption during pregnancy, folic acid supplement use, breastfeeding and current childhood body surface area. Analyses on microalbuminuria are Odds Ratio's

BSA, Body surface area

^a^eGFR_creat_: Estimated glomerular filtration rate calculated using the revised Schwartz 2009 formula [20]. GFR_Cys_: eGFR based on cystatin C levels according to the Zappitelli formula [21].

To investigate non-linearity, we created quintiles of fetal first trimester growth. Table 2 shows that as compared to the highest quintile of fetal first trimester crown–rump length, the lowest quintile was associated with a larger childhood combined kidney volume (difference 5.32 cm^3^, 95 % confidence interval 1.06–9.57), but not with eGFR and microalbuminuria. Sensitivity analyses using tertiles of fetal first trimester crown–rump length showed no associations with childhood kidney outcomes. Results of this sensitivity analyses are presented in ESM Table 3; observed data before multiple imputations are presented in ESM Table 2. The association of fetal first trimester crown–rump length with childhood kidney volume was not observed in non-imputed data; in addition analyses of non-imputed data showed no associations with other kidney outcomes in childhood. The lack of significant associations in non-imputed datasets may be due to the smaller numbers: analyses on imputed data were based on 1176 datasets, whereas analyses on the non-imputed data were based on 934 datasets.

## Discussion

In this population-based prospective cohort study, we evaluated the associations of fetal first trimester crown–rump length with kidney growth and function in childhood. We did not observe consistent associations of fetal first trimester growth with kidney outcomes in childhood.

Some methodological issues need to be addressed. We used a subgroup of a large population-based prospective cohort study to examine the kidney consequences of fetal first trimester growth restriction. Only mothers with a first trimester crown–rump measurement and a reliable first day of their last period were eligible for entry into the study, and women meeting these criteria represented only a small subgroup of the full study. We used the first day of the last menstrual period in women with a regular menstrual cycle to date gestational age since we could not measure the timing of ovulation and implantation. It is therefore possible that a misclassification of gestational age could have occurred [25]. Of the eligible subgroup, blood samples had been collected from 67 % of all children. Our results would be biased if the results differed between children with and without follow-up measurements at the age of 6 years. Although this possibility seems unlikely, we cannot exclude it. Children for whom no blood samples were available had a lower mean birth weight than their counterparts for whom blood samples were available. Our results would be biased if the associations of first trimester growth with childhood kidney function differed between children with and without blood samples. Since smaller numbers of blood samples were available in children with lower birth weight, our observed effect estimates may be underestimated. Glomerular number cannot be evaluated in vivo, but kidney size and glomerular number correlate in pathological studies in childhood and adulthood [26–28]. We estimated the GFR using the Schwartz formula [20], which is based on serum creatinine levels, and the Zappitelli formula [21], which is based on serum cystatin C levels; both formulas have been validated in pediatric populations. The eGFR was higher when calculated based on creatinine concentrations than on cystatin C concentrations, a difference which is in line with previous studies [29]. However, no difference in results were observed when we used eGFR based on creatinine concentrations compared to cystatin C concentrations, and we found no differences in outcomes between those formulas. It has been suggested that serum cystatin C levels may be the superior biomarker for evaluating kidney function compared to serum creatinine levels [30]. However, to date it is not clear which formula provides the best estimation of the GFR [30]. We used a random urine sample to determine the albumin to creatinine ratio to evaluate microalbuminuria. Since intra-individual variation in urinary albumin secretion may be large, the variability would probably have been lower if we collected first morning void samples [31]. Finally, although we adjusted for several potential confounders, residual confounding might still be a problem because of the observational design of the study.

Growth and development rates are higher in fetal life than in childhood. The highest rate of organogenesis in humans occurs during the first trimester. The first trimester may therefore be a critical period in terms of developing risk factors for diseases in later life. We adopted an approach used in previous studies focused on fetal first trimester growth in relation to birth outcomes and cardiovascular outcomes and used quintiles for fetal first trimester growth [12, 14]. No major differences in results were observed when we used tertiles of fetal first trimester growth instead of quintiles. Longitudinal studies, including studies based on the same cohort as that in the present study, have shown associations of impaired first trimester growth with increased risks of premature birth, low birth weight and being small for gestational age at birth [12, 32, 33]. Results from this cohort study have also previously shown that fetal first trimester growth restriction is associated with cardiovascular risk factors in childhood [14]. The analyses in this study were performed in a healthy population, with the majority of children born at term and normal weight for gestational age. To the best of our knowledge, no other human studies have evaluated the associations of first trimester growth with kidney function in later life. Nephrogenesis starts around week 5 of gestation, and the first nephrons begin to form at approximately week 9 [34]. Nephrogenesis continues during gestation and stops around week 34–36 [10]. Against this background, our aim was to identify the role of first trimester fetal development in kidney development.

Using data from the same cohort as that enrolled in the present study, we previously reported that lower fetal growth from second trimester onwards is associated with lower combined kidney volume and eGFR in childhood [8]. Also, a previous study among children born preterm showed that size for gestational age is correlated with kidney volume at the ages of 0, 3 and 18 months, which implies that impaired fetal growth has consequences for kidney growth in infancy [35]. Some studies on fetal growth impairment have shown a slight catch-up kidney growth postnatally in small for gestational age infants, but the results are inconclusive [35, 36]. The current study extends these previous findings since it is focused on first trimester of pregnancy, a period of which little is known in relation to kidney outcomes. We did not observe consistent associations of fetal first trimester growth with kidney outcomes in childhood.

Smaller kidneys with fewer nephrons will lead to compensatory glomerular hyperfiltration, which might be beneficial in the short term but can lead to glomerulosclerosis and impaired kidney function in later life [1]. Hyperfiltration might increase renal mass while glomerular number is relatively low [37]. However, it is not possible to distinguish hypertrophy or normal growth by ultrasonography. Also, it is not known when hypertrophy exactly occurs, and it is difficult to determine whether glomerular enlargement is caused by glomerular hyperfiltration [38, 39]. In the present study, we observed an inverse association between fetal first trimester crown–rump length and kidney volume in childhood, which we cannot fully explain. This finding is in line with the results of a previous study from the same cohort which showed that the lowest tertile of gestational age-adjusted abdominal circumference in the third trimester is associated with a larger relative fetal kidney volume [40]. Renal hypertrophy may be one explanation for this inverse association; however, more studies are needed to replicate our findings and to identify the underlying mechanisms.

We performed multiple statistical tests. However, since the kidney outcomes were highly correlated, we did not adjust analyses for multiple testing. Therefore, the observed association between the lowest first trimester crown–rump length and kidney volume should be interpreted carefully and may be a chance finding. Decreased fetal growth might lead to impaired kidney function by other mechanisms than smaller kidneys; for example, multiple studies have suggested that epigenetic changes in response to adverse fetal exposures lead to developmental adaptations [6, 41, 42].

In conclusion, we did not observe associations between fetal first trimester growth restriction and kidney size and function in childhood. These findings do not support the hypothesis that the first trimester is a critical period for kidney function in later life, and further studies on this hypothesis are needed.

## Electronic supplementary material

Below is the link to the electronic supplementary material.

ESM 1 (DOCX 45 kb)
