# Supplementary material for: Fetal first trimester growth is not associated with kidney outcomes in childhood
Source: Pediatr Nephrol. 2016 Oct 27;32(4):651–8. doi: 10.1007/s00467-016-3537-8 (PMC5334431; doi:10.1007/s00467-016-3537-8)
Supplement: Supplementary file 1 — (DOCX 45 kb) [file 467_2016_3537_MOESM1_ESM.docx]

**Supplementary material**

**Fetal first trimester growth is not associated with kidney outcomes in childhood**

Hanneke Bakker^1,2,3^; Romy Gaillard^1,2,3^ ; Albert Hofman ^2,4^, Irwin K. Reiss ^3^, Eric A. P. Steegers ^5^; Vincent W.V. Jaddoe*^1,2,3^

1. The Generation R Study Group, Erasmus University Medical Center, Rotterdam, The Netherlands

2. Department of Epidemiology, Erasmus University Medical Center, Rotterdam, The Netherlands

3. Department of Pediatrics, Erasmus University Medical Center, Rotterdam, The Netherlands

4. Department of Epidemiology, Harvard T.H. Chan School of Public Health, Boston, Mass, USA

5. Department of Obstetrics and Gynaecology, Erasmus University Medical Center, Rotterdam, The Netherlands

Content

Imputation procedure

Table S1. Maternal and child characteristics of subjects with and without blood samples

Table S2. Fetal First Trimester Growth Quintiles and Childhood Kidney Volume and Function: non-imputed data

Table S3. Fetal First Trimester Growth Tertiles and Childhood Kidney Volume and Function

Table S4. Correlations between kidney volume and kidney function

Table S5. Correlations between body surface area-related kidney volume and kidney function

Figure S1. Normal distribution of of BSA-related renal volume

**Imputation procedure**

To reduce the possibility of potential bias associated with missing data and to maintain statistical power, missing values were imputed using the multiple imputations procedure.(1) For the multiple imputations, we used Fully Conditional Specification, an iterative of the Markov Chain Monte Carlo approach. For each variable, the fully conditional specification method fits a model using all other available variables in the model as predictors, and then imputes missing values for the specific variable being fit. In the imputation model for the analyses focused on the associations of early growth outcomes with kidney outcomes in childhood, we included all covariates except childhood body surface area plus maternal weight gain during pregnancy and height and weight of the child aged 6. Furthermore, we added the determinants and outcomes studied in the imputation model as prediction variables only. Determinants and outcomes were not imputed themselves. Five imputed datasets were created and analyzed together. For the conditional analyses only, we additionally imputed fetal and childhood growth characteristics using a similar imputation model.

1. Sterne JA, White IR, Carlin JB, Spratt M, Royston P, Kenward MG, et al. Multiple imputation for missing data in epidemiological and clinical research: potential and pitfalls. BMJ (Clinical research ed. 2009;338:b2393.

**Table S1. Maternal and child characteristics in subjects with and without blood samples (N = 1,176)**

|  | **Subjects with blood samples**  **N = 794** | **Subjects without blood samples**  **N = 382** |
| --- | --- | --- |
| **Maternal characteristics** |  |  |
| Age, median (90% range), yr | 31.8 (23.1-38.4) | 31.6 (22.0-37.6) |
| Height, mean (SD,) cm | 168.8 (7.1) | 168.3 (6.8) |
| Pre-pregnancy weight, mean (SD), kg | 66.9 (11.8) | 67.2 (12.0) |
| Pre-pregnancy body mass index, kg/m2 | 23.4 (3.8) | 23.7 (4.1) |
| Parity, nulliparous, No. (%) | 467 (58.9) | 247 (64.7) |
| Ethnicity, No. (%) |  |  |
| European | 584 (73.6) | 268 (70.2) |
| Non -European | 209 (26.4) | 111 (29.3) |
| Educational level, No. (%) |  |  |
| No higher education | 344 (43.4) | 182 (47.6) |
| Higher education | 449 (56.6) | 200 (52.4) |
| Smoking, No. (%) |  |  |
| Non-smoking | 590 (74.4) | 292 (76.4) |
| Continued smoking | 206 (26.6) | 90 (23.6) |
| Folic acid supplement use, No. (%) |  |  |
| No use | 103 (13.0) | 42 (13.5) |
| First 10 weeks use | 239 (30.1) | 99 (31.8) |
| Preconception use | 451 (56.9) | 170 (54.70 |
| **Fetal characteristics** |  |  |
| Gestational age at fetal crown to rump length, median (90% range), weeks | 12.4 (10.6-13.9) | 12.4 (10.9-13.6) |
| First trimester fetal crown to rump length, mean (SD), mm | 61.3 (11.4) | 60.7 (11.7) |
| **Birth and infant characteristics** |  |  |
| Males, No. (%) | 392 (49.4) | 178 (46.6) |
| Gestational age, median, (90% range) weeks | 40.1 (37.1-42.0) | 40.2 (37.0-42.1) |
| Birth weight, g | 3,490.6 (536.8) | 3394.8 (572.0)** |
| Ever breastfeeding, % |  |  |
| No | 61 (7.7) | 27 (7.1) |
| Yes | 732 (92.3) | 355 (92.9) |
| **Child characteristics** |  |  |
| Age, (median 90% range), yr | 6.0 (5.7-7.0) | 6.0 (5.7-6.5)** |
| Height, mean (SD), cm | 119.2 (5.5) | 1.19 (5.6) |
| Weight, mean (SD), kg | 22.9 (3.6) | 22.7 (3.8) |
| Body mass index, mean (SD), kg/m^2^ | 16.0 (1.3) | 16.1 (1.8) |
| Kidney volume combined, cm^3^ | 120.1 (22.3) | N.A. |
| eGFR, (Schwartz, creatinine based) (ml/min per 1.73m²) | 119.4 (15.4) | N.A. |
| eGFR, (Zapitelli, cystatin C based), (ml/min per 1.73m²) | 102.8 (15.9) | N.A. |
| Microalbuminuria, No. % | 52 (6.7) | 30 (8.2) |

Values are means (standard deviation), median (90% range) or number of subjects (valid %).

eGFR, estimated glomerular filtration rate

N.A. not applicable

T-tests were used for continuous variables, chi-square tests for categorical variables

* p-value < 0.05 ** p-value < 0.01

**Table S2.** Fetal First Trimester Growth Quintiles and Childhood Kidney Volume and Function (N = 934)^1^ non-imputed analyses

| **CRL quintiles**  **in SDS** | **Combined kidney volume**  **(cm3)** | **GFRcreat**  **(ml/min per 1.73m²)** | **GFR cys C**  **(ml/min per 1.73m²)** | **Micro albuminuria**  **(mg/mmol)**  **(OR)** |
| --- | --- | --- | --- | --- |
| 1 | 3.28 (-0.91, 7.47) | -0.32 (-4.63, 4.00) | 0.08 (-4.89, 5.04) | 0.53 (0.18, 1.54) |
| 2 | -3.52 (-7.70, 0.66) | -2.06 (-6.36, 2.25) | -3.43 (-8.39, 1.52) | 0.94 (0.38, 2.34) |
| 3 | 0.51 (-3.73, 4.76) | -0.80 (-5.08, 3.49) | 0.85 (-4.07, 5.77) | 0.99 (0.39, 2.52) |
| 4 | 1.80 (-2.47, 6.06) | 0.12 (-4.33, 4.57) | -0.84 (-5.97, 4.28) | 1.12 (0.44, 2.85) |
| 5 | *reference* | *reference* | *reference* | *reference* |
| p-value  for trend | 0.69 | 0.38 | 0.79 | 0.45 |

**^1^**Values are regression coefficients (95% confidence interval) that reflect the difference in childhood kidney outcomes between first-trimester crown to rump length fifths, highest fifth is reference group. Model is adjusted for duration of last menstrual cycle, and child sex and age at outcome measurements, maternal age, educational level, ethnicity, parity, pre-pregnancy body mass index, smoking during pregnancy, alcohol consumption during pregnancy and folic acid supplement use, breastfeeding and current childhood body surface area.

**Table S3.** Fetal First Trimester Growth Tertiles and Childhood Kidney Volume and Function (N = 1,176)^1^ non-imputed analyses

| **CRL quintiles**  **in SDS** | **Combined kidney volume**  **(cm3)** | **GFRcreat**  **(ml/min per 1.73m²)** | **GFR cys C**  **(ml/min per 1.73m²)** | **Micro albuminuria**  **(mg/mmol)**  **(OR)** |
| --- | --- | --- | --- | --- |
| 1 | 2.28 (-0.55, 5.10) | -0.45 (-3.20, 2.30) | -1.58 (-4.52, 1.36) | 0.80 (0.43, 1.48) |
| 2 | *Reference* | *Reference* | *Reference* | *reference* |
| 3 | 0.02 (-2.85, 2.89) | 0.24 (-2.51, 2.99) | 0.03 (-2.91, 2.98) | 1.15 (0.64, 2.07) |
| p-value  for trend | 0.11 | 0.58 | 0.83 | 0.51 |

**^1^**Values are regression coefficients (95% confidence interval) that reflect the difference in childhood kidney outcomes between first-trimester crown to rump length fifths, highest fifth is reference group. Model is adjusted for duration of last menstrual cycle, and child sex and age at outcome measurements, maternal age, educational level, ethnicity, parity, pre-pregnancy body mass index, smoking during pregnancy, alcohol consumption during pregnancy and folic acid supplement use, breastfeeding and current childhood body surface area.

**Table S4.** Correlations between kidney volume and kidney function

|  | **Combined kidney volume**  **(cm^3^)** | **eGFRcreat**  **(ml/min per 1.73m²)** | **eGFRcys C**  **(ml/min per 1.73m²)** |
| --- | --- | --- | --- |
| **Combined kidney volume (cm^3^)** | 1 | - | - |
| **eGFRcreat (ml/min per 1.73m²)** | 0.23** | 1 | - |
| **eGFRcys C (ml/min per 1.73m²)** | 0.10** | 0.29** | 1 |

**Table S5.** Correlations between body surface area-related kidney volume and kidney function

|  | **BSA-related**  **kidney volume**  **(cm^3^)** | **eGFRcreat**  **(ml/min per 1.73m²)** | **eGFRcys C**  **(ml/min per 1.73m²)** |
| --- | --- | --- | --- |
| **BSA-related kidney volume (cm^3^)** | 1 | - | - |
| **eGFRcreat (ml/min per 1.73m²)** | 0.28** | 1 | - |
| **eGFRcys C (ml/min per 1.73m²)** | 0.13** | 0.29** | 1 |

**Figure S1.** Normal distribution of of BSA-related renal volume


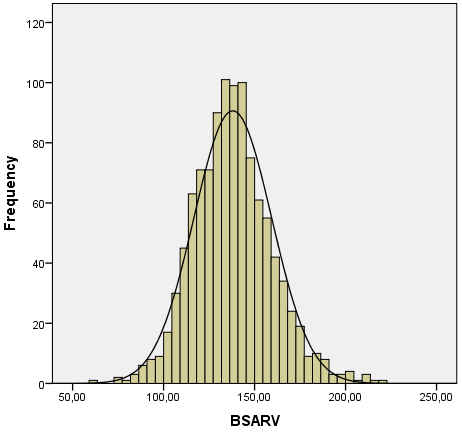


Mean = 138.01

Standard deviation = 21.41

BSARV in cm3/m2
